# Short-term pre-meal whey protein microgel supplementation reduces postprandial glycemia and appetite in adults with overweight: An open-label randomised controlled trial

**DOI:** 10.1016/j.obpill.2025.100183

**Published:** 2025-05-26

**Authors:** Ian J. Neeland, Kostas Tsintzas, Bo Ahrén, Robert J. Chilton, Ambra Giorgetti, Alric Mondragon, Rachel Ambiaux, Eugenia Migliavacca, David Philippe, Olivier Aprikian, Odd Erik Johansen

**Affiliations:** a Harrington Heart and Vascular Institute, University Hospitals Cleveland Medical Center, and Case Western Reserve University School of Medicine, Cleveland, OH, 44106, USA; b MRC Versus Arthritis Centre for Musculoskeletal Ageing Research, School of Life Sciences, University of Nottingham Medical School, Queen's Medical Centre, Nottingham, NG7 2UH, UK; c Department of Clinical Sciences, Lund University, 221 00, Lund, Sweden; d University of Texas Health Science Center, San Antonio, TX, USA; e Societe de Produits Nestlé, 1000, Lausanne, Switzerland; f Nestlé Health Science, 1000, Lausanne, Switzerland; g Nestlé Health Science, Bridgewater, NJ, 08807, USA

## Abstract

**Background:**

Premeal whey protein (WP) consumption may reduce postprandial glucose (PPG) levels and appetite. We assessed the effects of twice daily consumption of a low-dose non-gelling novel WP formulation (WP microgel [WPM]) on PPG, self-reported appetite, and ad-libitum food consumption.

**Methods:**

This was a randomized, prospective, open-label, controlled, single-center crossover study, and adults with BMI 27–35 kg/m^2^ were randomized to consume either 125 mL of 10 g WPM or control (water) 15 min before breakfast and lunch for four consecutive days. Three days were under free-living conditions, and the 4th day was at the clinic where breakfast (09:00 a.m.) was standardized (323 kcal, 7.0 g proteins), and lunch (12:00 p.m.) ad-libitum (pizza, 228.8 kcal/9.9 g proteins per 100 g). Following a 3-day wash-out, participants were switched to the opposite regimen. The primary confirmatory endpoint was breakfast 2 h-PPG (assessed using CGM) analyzed as iAUC_-15-120min_ using a linear mixed effects model. Appetite was captured by frequent self-reporting (hunger, desire, amount, fullness, satisfaction) using a visual analogue scale (0–100 mm). Ad-libitum food consumption (lunch) was assessed by weighing the amount consumed.

**Result:**

18 individuals (8 females, median age 57 years, BMI 29.8 kg/m^2^, HbA1c 5.5 %) were randomized and consumed products. The breakfast 2 h-PPG iAUC was 39.3 % lower with WPM compared with control (LSM iAUC Ratio WPM/control (95 % CI): 0.607 [0.4 43, 0.831], p = 0.0047), and during lunch numerically reduced (p = 0.0649). Appetite scores during breakfast and lunch supported a modest suppressing effect of the WPM. Food consumption during the ad-libitum lunch was significantly reduced by 9.4 % (WPM vs Control −66.8 kcal [-133.1, −0.6], p = 0.0482).

**Conclusions:**

A 125 mL pre-meal dose of WPM consumed twice daily before breakfast and lunch for 4 days in adults with obesity significantly reduced breakfast PPG and had a moderate appetite-suppressing effect, which led to a significantly lower energy consumption during ad-libitum lunch (NCT06593769).

## Graphical abstract

**Figure:**
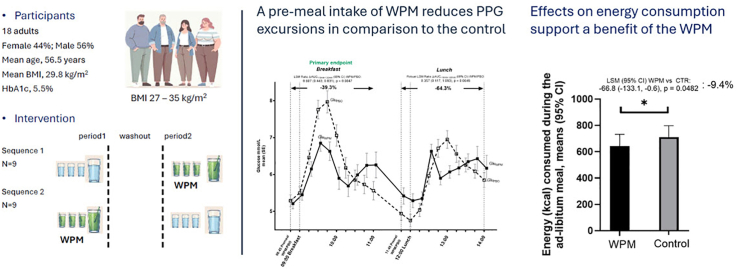

## 1 Introduction

Whey proteins (WPs) are found in dairy products, and one of the most notable early metabolic clinical effects observed by consuming WP, in particular as a pre-meal intervention, was a consistent lowering of post-prandial glucose (PPG) excursion. This has been observed across a range of background characteristics, like normo- or hyperglycaemia, high or low body mass index (BMI), age, and, sex [[1], [2], [3], [4]]. This effect has been ascribed to the ability of WP to stimulate the secretion of incretin peptides, such as glucagon like-peptide 1 (GLP-1) and glucose-dependent insulinotropic polypeptide (GIP) [[2], [3], [4]], augmenting the release of insulin, and slowing of the rate of gastric emptying (GE). Reducing PPG contributes to reducing the overall glycemic burden, which appears to be particularly important in prediabetes, gestational diabetes mellitus, and early stages of type 2 diabetes (T2D), where the relative contribution of PPG, as compared with fasting glucose, to overall glucose burden reflected by HbA1c level, is more clinically important [5]. WP could therefore be an adjunct to any intervention to support managing glucose levels, e.g., through lifestyle or pharmaceutical interventions.

WPs have also been associated with a beneficial effect on appetite [[6], [7], [8], [9]], e.g., they may reduce hunger and increase feelings of fullness – as observed in single dose investigations [[6], [7], [8]] as well as following longer-term dosing up to 12 weeks [9]. This has been proposed to be related to the reduction in gastric emptying, which is well-described being linked with feelings of fullness and satiety [10], a moderate increase in GLP-1 levels, and specific effects of the bioactive peptides or the amino-acid composition found in WP [11]. This particular effect of WPs may be of specific relevance for people who are trying to lose weight, or are in a weight-maintenance phase, which can be further aided by the WP support for lean mass and muscle mass maintenance [12].

However, one drawback with traditional WPs has been the need to ingest these well in advance of a meal (usually 30 min or more), and in a relatively high dose (typically more than 25 g), which adds calories [2], since each gram of protein provides ∼4 kcal. To illustrate, a meta-analysis suggested that one would require consumption of 20–72 g of WP per meal occasion to achieve significant effects, where the most pronounced effects were associated with higher amounts consumed [9].

New technologies have emerged that could address these practical hurdles, e.g., shorten the time of ingestion before a meal and lower the caloric impact. One such new technology that could enable use of a lower dose, and allow ingestion closer to the meal, is a new WP formulation (WP microgel [WPM]) [3], developed as a specific form of WP aggregate, delivered in a low-dose (10 g), but highly concentrated, ready-to-drink (RTD) solution. In healthy individuals with overweight or obesity [13], as well as in people with T2D [3], this novel formulation was shown to acutely reduce PPG; and specifically in people with T2D, also shown to increase GLP-1 levels [3]. However, there are no dedicated studies conducted with longer term or multiple daily dosing of this product, nor has it been tested specifically with the aim to assess effects on appetite and food consumption.

Herein we tested if 10 g WPM consumed 15 min before breakfast and lunch for four consecutive days could provide clinically meaningful effects on PPG, mean 24 h glucose values, self-reported satiety, and total food consumption during an ad-libitum lunch, involving people living with overweight or obesity, without T2D.

## 2 Methods

### 2.1 Study design and participants

This was a randomized, prospective, open-label, controlled, single-center crossover study (ClinicalTrials.gov: NCT06593769) that recruited adults (men and women) living with overweight or obesity, but without T2D, that at the time of enrolment to the study were not taking any medications for glycaemic or weight management. This study was designed to evaluate the effects of twice daily consumption of 125 mL pre-meal RTD solution containing 10 g WPM compared with an isovolumetric metabolically inert control (CTR; water) on PPG, mean 24 h glucose values, satiety and food consumption (Supplementary Fig. S1). The study protocol was approved by the «Commission cantonale d’éthique de la recherche sur l’être humain du Canton de Vaud», with reference number 2024-00731, and the study was carried out in compliance with the Harmonized Tripartite Guideline for Good Clinical Practice from the International Conference on Harmonisation [14] and the Declaration of Helsinki [15]. Participants provided written, informed consent.

The key eligibility criteria for participants were: BMI 27–35 kg/m^2^, age 45–70 years, and being in general good health (from medical history). Participants were not eligible if they had any past or ongoing medical or surgical condition (i.e., T2D, malignancy, gastrointestinal disease), or any known inborn errors of amino acid and protein metabolism. Known or suspected allergies or intolerances to any of the ingredients of the nutritional formulation (i.e., milk, lactose) were also exclusions. Furthermore, people who had random plasma glucose ≥ 11.1 mmol/L or fasting plasma glucose ≥ 7.0 mmol/L (finger-prick point-of-care testing), or HbA1c ≥ 6.5 % (finger-prick point-of-care testing) were excluded; any such results identified at screening would trigger an advice to seek a consultation with their general physician for further evaluation. The full list of inclusion and exclusion criteria is provided in the Supplementary Table S1.

### 2.2 Investigational products

The pre-meal drink of 10 g of WP was prepared with a novel technology aimed at increasing the WP concentration in a liquid matrix that did not gel, using a proprietary micelle-technology by generating microgels (WPM) [16]. The technology behind the formation of the novel concentrated WPM involves several steps, including heat treatment, and pH adjustment of native WP [16]. The active product was produced from a native whey protein isolate, at the Nestlé Bridgewater Pilot Plant (1007 US Highway 202/206 Bldg JR2, Bridgewater, NJ 08807, USA). The WPM was fully diluted in 125 mL water, without any evidence of gelling, and produced a palatable, RTD beverage (production batch number 1207429) with a shelf life of 12 months and being stable at both room temperature and in chilled environmental conditions. The product is available on the US market under the BOOST® brand. The total protein content was 9.5 % with a whey-protein component of 10 g per 125 mL RTD beverage and contained no fat, and residual carbohydrates (< 1.0 %; 50 kcal per 125 mL). The matching comparator was 125 mL of water. The reason for selecting water as a comparator was based on its ease of preparing an isovolumetric comparator for the RTD protein beverage, and its metabolically inert properties, which was important for the study objective of assessing glycemic and satiety effects.

### 2.3 Study procedures

All participants attended the clinic on five separate occasions (screening (V0), familiarization visit (V1), initiation visit (V2), and two endpoint assessment visits (V3 and V4).

Following a successful screening visit, where also general safety laboratory tests were drawn to determine eligibility, a familiarization visit V1 followed, where additional instructions were given, and an initiation visit (V2), where people were introduced to the study procedures (product consumption, standardized breakfast and ad-libitum lunch meal, and reporting of satiety using a visual analogue score [VAS]). Additionally, they all received a continuous glucose monitor (CGM) and a sensor (masked for participants), that was intended to be kept for the full duration of the study (i.e., for 12 days, and until approximately 2 h after lunch had been consumed on V4). The CGM used in this study was FreeStyle Libre Pro IQ (Abbott Diabetes Care Ltd., Witney, Oxfordshire, OX29 OYL, UK) using the IQ sensor (blinded CGM). The system was applied by study staff to the underside of the participant's upper arm to measure individual interstitial glucose levels in 15-min intervals. At the conclusion of the study, all CGMs were collected and data analyzed according to a prespecified plan. All participants were instructed to get in touch with the site as soon as possible in case the device fell off, for replacement.

Any individual that had completed the 3-day CGM assessment during the “control as 125 mL pre-meal RTD beverage” phase would be notified in writing to get in touch with their general practitioner for assessment of potential prediabetes if the 24 h average glucose value from the CGM during the 72 h-period exceed 6.6 mmol/L. Although there are currently no CGM-based criteria for diabetes or prediabetes diagnosis; this advice is based on observations from literature [17], and a study that proposed the normative reference value in healthy people for 24 h mean blood glucose value from CGM of ≤ 6.6 mmol/L (119 mg/dL) [18].

Following the conclusion of V2, people left the clinic and embarked on a 3 day free-living condition phase. This was followed by an endpoint assessment visit (V3). V3 was followed by 3-days of wash-out, before participants were crossed to another 3-days of free-living conditions where participants consumed the products 15 min ahead of breakfast and lunch, before arriving at the clinic for the second endpoint assessment at V4. To standardize the physiologic conditions ahead of the clinic visits, participants were encouraged to not change any lifestyle or eating habits during the 3-days of free-living conditions.

At V3 and V4 participants were asked to arrive in the morning after an overnight fast. Here they would consume the study products 15 min ahead (at 07:45) of a standardized breakfast meal (at 08:00) consisting of 2 slices of white bread, 20 g of jam, and 250 mL glass of orange juice (total kcal: 323; proteins 7.0 g, carbohydrates 68.2 g (sugars 34.1 g), fat 9.5 g (saturated fat 0.5 g)), and before (at 11:45) the ad-libitum lunch meal at 12:00 (Pizza Margherita, Hiestand Schweiz AG; per 100 g: 228.8 kcal, proteins 9.9 g, carbohydrates 34.2 g (sugars 0.5 g), fat 4.7 g (saturated fat 2.5 g)). The amount of food that was consumed during the ad-libitum pizza meal was assessed by weighing the amount provided and the amount that was left-over.

### 2.4 Primary confirmatory endpoint

The study hypothesis was that four days of twice daily consumption of WPM 15 min before meals compared to similar volume of a control product (water) would lead to reductions in PPG level. The approach to test this hypothesis was to compare the 2 h PPG response (on CGM) after a standardized breakfast on day 4 between interventions. The 2 h timepoint was defined based on design of previous experiments [3], and the conventional agreement of this being a reasonable timepoint for when glucose levels return to baseline following a mixed meal challenge in healthy people. The glucose values were captured using CGM and were allocated to 15 min time intervals coinciding with t = −15 min (start of consumption of premeal beverage), t = 0 (start of breakfast), and thereafter at t = 15, 30, 45, 60, 90, and 120 min. This was a predefined confirmatory endpoint (Supplementary Table S2), where the primary analysis was defined as the 2 h incremental area under the curve (iAUC).

### 2.5 Secondary glycemic endpoints

The predefined secondary glycemic endpoints were mean 24 h glucose from the 3-days of free-living conditions, as well as the mean glucose during the time-window that captured the meal occasions where the premeal products were consumed (i.e., 08:00–16:00 as an estimate that covered the PPG of the breakfast and lunch meals).

### 2.6 Secondary appetite endpoints

The hunger/fullness VAS questionnaires used in the study consisted of 5 questions measured on a semi-anchored 0–100 mm scale [19]. The participants was asked to answer the questionnaire at the time of the pre-meal intervention consumption (15 min before the start of meals), at the start of breakfast, and every 30 min thereafter until around 2 h after lunch during the visit days. The following dimensions were assessed: 1) “How hungry do you feel?”, 2) “How strong is your desire to eat?”, 3) “How much do you think you could eat?”, 4) “How satisfied do you feel?”, and 5) “How full do you feel?”. These were labelled with anchors such as “not at all” at 0 mm and “extremely” at 100 mm. Participants were asked to place a single mark on the horizontal bar which will best describe their feeling for each question. Ratings were manually scored using a ruler and measured to the nearest millimeter. A combined appetite composite (CAC) score was calculated as the sum of the scores from the 5 dimensions, with dimension 4 and 5 scored inversely (i.e., 100 – score).

The mean score, iAUC, and the tAUC, for the 5 VAS scores and the CAC, and individual timepoints, were calculated for the breakfast meal and the lunch meal separately. For the breakfast meal, the score at 15 min before breakfast was used as the baseline, i.e., immediately before consumption of the first preload (i.e., at 08.45 a.m./t = −15 min-breakfast), and scores were collected thereafter immediately before breakfast (09.00 a.m./t = 0-breakfast min), and every 30 min until immediately before the second preload at 11.45 a.m. (t = 165 min-breakfast/t = −15 min-lunch). For the lunch meal, the baseline was the scores immediately before consumption of the second preload at 11.45 a.m. (t = 165 min-breakfast/t = −15 min-lunch), and then scores were assessed immediately before lunch (12.00 p.m./t = 0-lunch min), and then every 30 min for 2 h after lunch (i.e., until 2.00 p.m.). For VAS dimensions 1–3, the mean, iAUC, and tAUC were estimated as the sum of the areas located below the baseline value. For the VAS dimensions 4 and 5, however, it was estimated based on the areas located above the baseline value.

Food consumption during the ad-libitum lunch meal was assessed by weighing the amount of food provided and the left-over, and then subtracting the 2 amounts. This was then converted to kcal based on the nutritional declaration of the product, where 100 g of the food product provides 228.8 kcal.

### 2.7 Safety and adverse events (AEs)

Occurrence of AEs was proactively assessed by queries at all visits post screening, and all AEs (spontaneously reported or inquired, as well as those observed) during the course of the study were captured, and were summarized descriptively. AEs were coded using Medical Dictionary for Regulatory Activities (MedDRA), Version 25.0.

### 2.8 Randomization, and sample size considerations

Randomization occurred in a 1:1 ratio to either sequence WPM/Control or Control/WPM on visit day 1 according to the crossover design, without stratification. Patients assigned to the first sequence with WPM beverage or control, received control and WPM beverage, respectively, during second sequence. Randomization was carried out using Medidata Rave RTSM.

The study hypothesis was that a low dose of WPM given 15 min before a standardized breakfast would lower the PP blood glucose levels compared to control in individuals with overweight or obesity, without T2D. We derived that a total of 15 participants (completers) would be required to detect an effect size of 0.56, representing a reduction of 20 % in incremental AUC over 2 h [3], following the standardized breakfast (iAUC-15-120min) between WPM and control (water), based on a within-subject correlation of 0.8, and a power of 90 % at the 2-sided 5 % significance level. Additionally, assuming a roughly 20 % drop-out rate, 18 participants were planned for enrolment.

### 2.9 Analysis

Depending on the distribution of data, patient characteristics were described using median (min, max), mean ± standard error, or mean (min, max) for continuous variables and proportions for categorical variables. Descriptive data for graphical presentation are shown as mean (standard error).

All the statistical analyses were performed using R 4.4.1.

Comparisons were performed by comparing mean changes in iAUC between the intervention and control groups over the different time periods using mixed effect model with intervention arm and period (period 1 and period 2) and a relevant baseline measurement as fixed effects and subject as random effect (lmer function from the lmerTest package in R). Results are expressed as effect estimates (LSmeans) with a 95 % confidence interval (CI) (emmeans R package). The 2 h PPG response following breakfast served as the primary confirmatory endpoint and was analyzed as iAUC-15-120 min using a linear mixed effects model. This model included baseline glucose levels, treatment, and period as fixed effects, while all participants from the Full Analysis Set (FAS) population were treated as a random effect. The model was estimated using Restricted Maximum Likelihood (REML), which provides unbiased estimates of variance components in mixed-effects models. Additionally, the degrees of freedom for the fixed effects were calculated using Kenward-Roger's method.

To evaluate the effect of the intervention at each time points for each of the hungry/appetite VAS scores and the CAS, a linear mixed-effects model was fitted using the lme function (from the nlme package in R). The model includes as fixed effects: the relevant baseline value, period, treatment group, and timepoints, as well as the interaction between treatment and timepoints to assess whether the treatment effect varies over time; as random effect: subjects to account for individual-level variability, acknowledging that repeated measures are nested within subjects. An unstructured correlation matrix (corSymm) was used to model the within-subject correlation across different timepoints. The varIdent function was used to allow for heterogeneous residual variances across timepoints, addressing unequal variances observed at different timepoints. The model is estimated using REML. The nlme::lme function utilized the containment method for computing degrees of freedom for fixed effects.

Relative difference between treatment groups was calculated in % as estimated treatment difference/estimated mean for placebo × 100 %. A two-sided nominal *p*-value < 0.05 was conventionally considered significant, and there was no correction for multiple testing.

## 3 Results

In total, 26 individuals were screened (Oct–Nov 2024), of whom 18 (8 women) were randomized and consumed products, and represent the intention to treat analysis set. One participant discontinued after the first sequence (on control) and all the others completed two sequences of product consumption according to the randomization scheme (consort diagram in Supplementary Fig. S3). Overall, the median age was 56.5 years, HbA1c 5.5 %, and BMI 29.8 kg/m^2^, respectively (Table 1).

**Table 1:** Baseline characteristics of the 18 participants with overweight or obesity in the study. *n* (%) or median (min, max).

Parameter/Characteristic	Value
Sex	
Women	8 (44 %)
Men	10 (56 %)
Age, years	56.5 (45.0, 70.0)
Body Mass Index, kg/m^2^	29.8 (27.0, 31.6)
HbA1c, %	5.5 (4.7, 5.9)
HbA1c, mmol/mol	36.9 (27.3, 41.1)
Fasting plasma glucose, mmol/L	5.4 (4.5, 6.4)
Total cholesterol, mmol/L	4.7 (3.6, 5.7)
Low-density lipoprotein cholesterol, mmol/L	1.2 (0.7, 1.5)
Triglycerides, mmol/L	0.9 (0.5, 2.3)
Aspartate aminotransferase, U/L	18 (12, 31)
Alanine aminotransferase, U/L	16 (12, 24)
Creatinine, μmol/L	61 (47, 103)

### 3.1 Effects on glucose trajectories

**Fig. 1:**
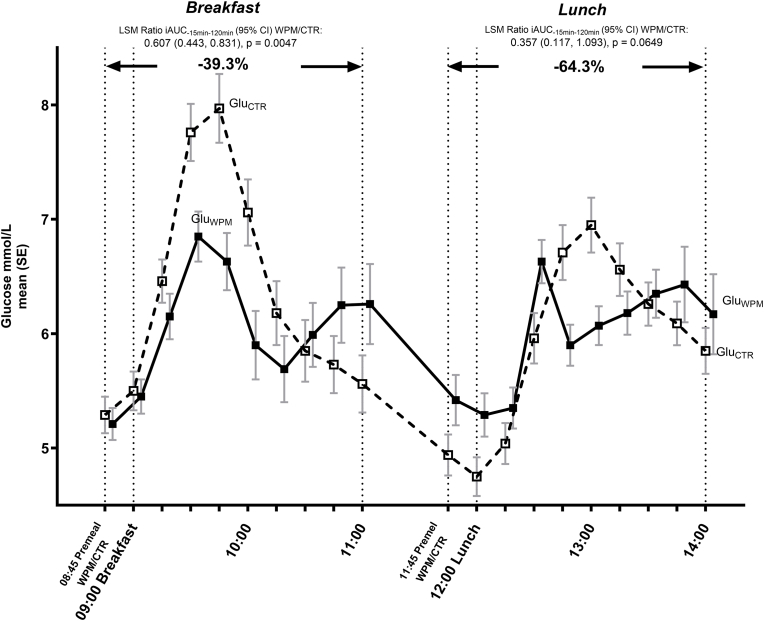
Glucose trajectories from 08:45 until 14:00 in people with overweight or obesity. WPM/CTR was consumed twice during this period, at 08:45 as a premeal drink before standardized breakfast, and at 11:45 as a premeal drink ahead of an ad-libitum lunch. Abbreviations: Glu—glucose, iAUC—incremental area under the curve, CTR—control, WPM, whey protein microgel, SE−standard error, h—hours.

Following four consecutive days of twice daily consumption of the 125 mL pre-meal WPM drink/control, the WPM significantly reduced the primary endpoint (2 h PPG iAUC during standardized breakfast on day 4) (Fig. 1) compared with control, by 39.3 % (LSM ratio (95 % CI) iAUC_−15min–120min_ WPM/CTR ratio 0.607 (0.443, 0.831), *p* = 0.0047).

**Fig. 2:**
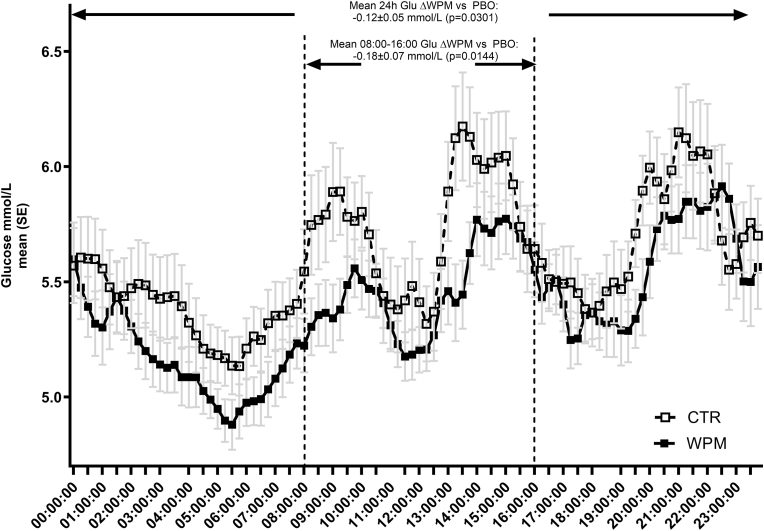
24 h glucose CGM trajectories during the 3 day free-living period in people with overweight or obesity. WPM/CTR was consumed twice daily during this period, 15 min before breakfast and 15 min before lunch. Abbreviations: Glu—glucose, CTR—control, WPM, whey protein microgel, SE−standard error.

A similar pattern of a reduction in 2 h PPG iAUC during the ad-libitum lunch was seen, but here, the difference did not meet statistical significance, likely owing to a high intra-person variability, as well the differences in baseline glucose levels between the groups at start of lunch (Fig. 1), which was not unexpected, and the lower amount of food consumed. Of note was the observation of a significantly lower mean 24 h glucose with WPM vs CTR during the three days under free-living conditions (Fig. 2), with a magnitude of difference of −0.12 ± 0.05 mmol/L (WPM 5.48 ± 0.11 mmol/L vs CTR 5.60 ± 0.11; p = 0.0301), as well as a significantly lower mean glucose during the period assumed to cover the meal occasions (breakfast and lunch) that involved the two pre-meal consumptions of WPM or CTR (i.e., 08:00–16:00), with a difference of −0.18 ± 0.07 mmol/L (p = 0.0144).

### 3.2 Effects on satiety parameters

**Fig. 3:**
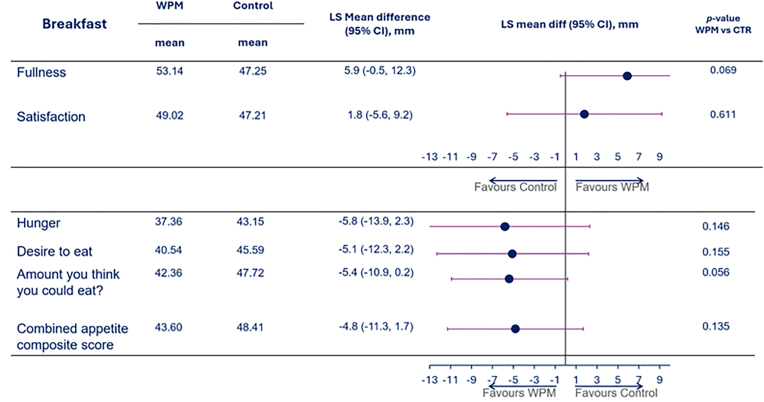
Mean VAS appetite scores across the various dimensions and the combined appetite composite score in people with overweight or obesity for the standardized breakfast when WPM/CTR was consumed 15 min ahead of start of the meal. Abbreviations: WPM, whey protein microgel, SE−standard error, CTR — control, LS – least square, CI - confidence interval.

**Fig. 4:**
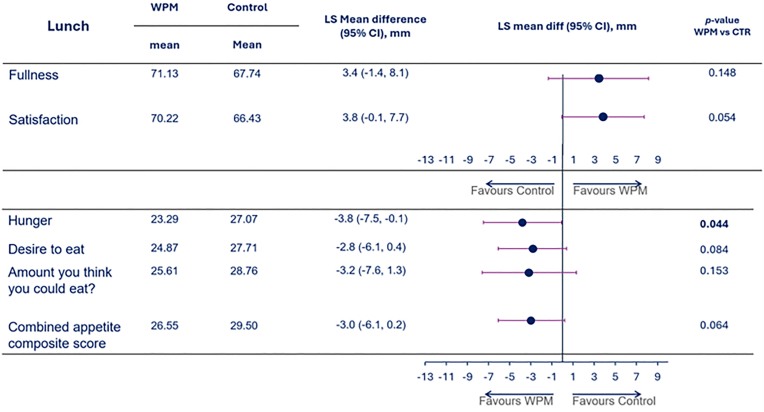
Mean VAS appetite scores across the various dimensions and the combined appetite composite score in people with overweight or obesity for the ad-libitum lunch meal when WPM/CTR was consumed 15 min ahead of start of the meal. Abbreviations: WPM, whey protein microgel, SE−standard error, CTR — control, LS – least square, CI - confidence interval.

The self-reported satiety scores during the in-clinic visits during the standardized breakfast and ad-libitum lunch, suggested that the premeal WPM compared to CTR influenced these in a direction of becoming less hungry and feeling fuller, however, there were heterogeneity in the results depending on meal occasion (breakfast vs lunch), and due to a high intra-individual variability. Therefore, although nominally the scores favored WPM, most comparisons did not meet statistical significance. Fig. 3, Fig. 4 depicts e.g., the mean VAS scores covering the entire breakfast (Fig. 3) and lunch (Fig. 4), and as indicated, despite all point-estimates favoring WPM, only the VAS score for “How hungry do you feel?” for the lunch meal was significantly lower with WPM by the conventional p < 0.05, while several reached borderline significance, e.g., during breakfast (Feeling of fullness (p = 0.069) and amount you think you could eat (p = 0.056)) and lunch (satisfaction (p = 0.054), desire to eat (p = 0.084), CAC score (p = 0.054)).

**Fig. 5:**
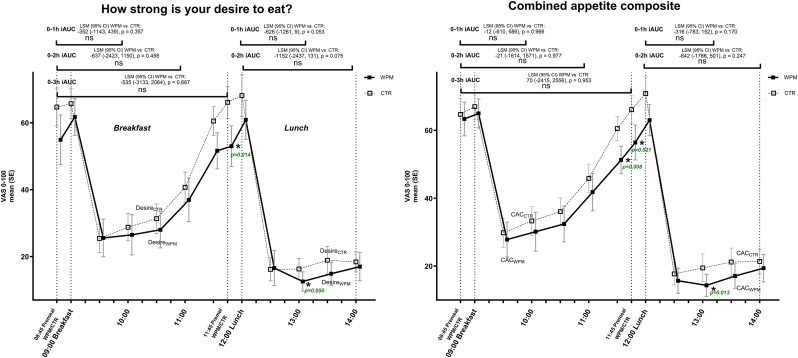
VAS trajectory for “How strong is your desire to eat” (left) and “Combined appetite composite (CAC)” score” (right) for the breakfast and lunch meals. Assessment of iAUC for 0–1 h, 0–2 h and 0–3 h are indicated for the breakfast meal, and iAUC 0–1 h and 0–2 h for the lunch meal at the top of the graph. Data in green indicate where there were significant differences for distinct timepoint comparisons. Abbreviations: WPM, whey protein microgel, SE−standard error, CTR — control, CAC – combined appetite composite, LS – least square, CI - confidence interval. ∗: p ​< ​0.05.

The same pattern was also true for the iAUC analysis of the six VAS scores for the two meal occasions (Fig. 5, Supplementary Figs. S4–S7). Here we observed borderline lower iAUC for dimensions “Desire to eat” (Fig. 5) during the ad-libitum lunch meal for the 1 h iAUC (p = 0.053) and 2 h iAUC (p = 0.075). Analyzing the tAUC responses provided a similar picture, although some comparisons between WPM and CTR also were significant (How hungry do you feel?: 1 h tAUC lunch (p = 0.015): 17.7 %; 2 h tAUC lunch (p = 0.033): 15.3 % and CAC score: 1 h tAUC lunch (p = 0.049): 11.2 %; 2 h tAUC lunch (p = 0.047): 11.2 %). When exploring individual timepoint comparisons for the VAS scores, we additionally observed significant differences between WPM and CTR for several timepoints/dimensions (i.e., Hunger (three occasions); Desire to eat (two occasions); Amount you can eat (one occasion); Fullness (two occasions); Composite score (three occasions)), whereas directional benefits were seen consistently at all timepoints (Fig. 5, Supplementary Figs. S4–S7).

### 3.3 Effects on food consumption

**Fig. 6:**
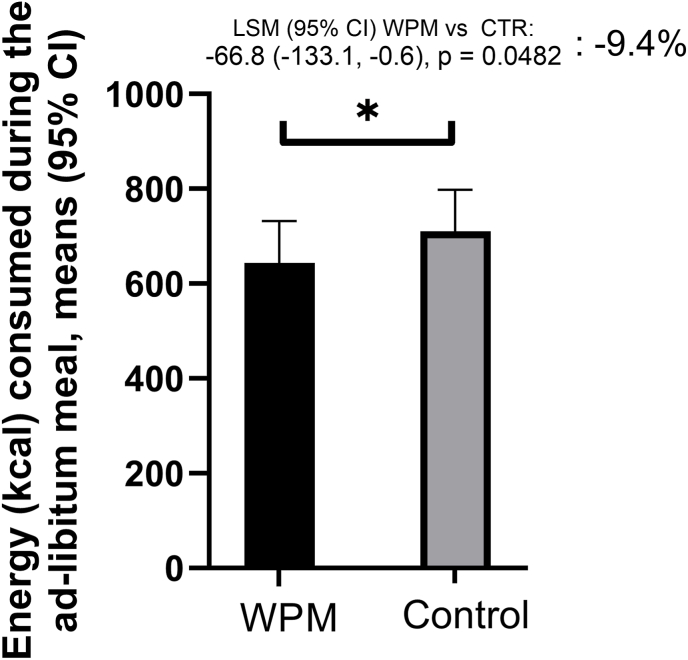
Energy consumption during ad-libitum lunch pizza meal by intervention group (WPM and CTR). Abbreviations: WPM, whey protein microgel, CTR – control. Values are LS means (95 % CI).

A lower amount of food was consumed with the WPM compared to CTR during the ad-libitum pizza meal serving at the clinic (Fig. 6), with a difference in energy (kcal) vs. CTR of −66.8 (−133.1, −0.6), p = 0.0482).

### 3.4 Adverse events

In total eleven adverse events occurred in a total of seven participants, which all were mild or moderate (Supplementary Table S3). Seven of these occurred in three subjects during the WPM consumption period. One AE (varicose vein) that occurred during the first treatment sequence with CTR led one participant to discontinue study participation.

## 4 Discussion

In individuals with overweight or obesity, without T2D, a highly concentrated RTD pre-meal formulation of a low dose of WP (10 g) provided as WPM in a 125 mL solution, shortly (15 min) ahead of breakfast and lunch for four consecutive days, significantly reduced breakfast PPG and average 24 h glucose levels, and a had modest appetite suppressing effect, which was associated with reduced food consumption during an ad-libitum lunch meal, when compared with a 125 mL pre-meal solution that was metabolically inert.

These results expand on previous observations for this particular WPM formulation that until now only had been tested in acute single dose interventions [3,13]. A notable difference is that the impact on 2 h PPG appeared to be more robust, −39 %, in the population studied (age 57 years, HbA1c 5.5 %, BMI 29.8 kg/m^2^), vs −22 % and −25 %, respectively in acute studies involving either people with T2D and overweight or obesity (age 62 years, HbA1c 7.5 %, BMI 29.2 kg/m^2^) [3], or relatively healthy people with overweight or obesity (age 49 years, BMI 31.2 kg/m^2^) [13]. The larger effect size may be ascribed to the twice daily dosing, as well as the longer-term dosing, albeit we acknowledge that four days is still relatively short.

These results are also notable in the context of a study where 15 g WP was consumed thrice daily (i.e., a total of 45 g/day) for 7 days involving 18 people with T2D (age 50 years, HbA1c 7.4 %, BMI 33.3 kg/m^2^) where 2 h PPG was reduced by −24 % vs placebo [20]; which is also less than what we observed, and suggests that the WPM may be a metabolically more potent PP nutritional modulator, also given the difference in dose between the two studies (20 g vs 45 g per day). Of note, that same study over 7 days with 45 g WP exposure reduced mean 24-h blood glucose concentrations by 0.6 ± 1.2 mmol/L (p = 0.045) during WP compared with placebo (8.9 mmol/L vs 9.5 mmol/L) [20], which is a larger reduction than what we observed. It can be argued that this is likely related to the differences in baseline glycaemic levels, since participants in our study were all without T2D (HbA1c 5.5 %), whereas all had T2D in the study by Smith et al. (HbA1c 7.4 %) [20], given that intervention effects on reducing glycaemic levels depend on baseline values [21]). Other studies using WP in populations without T2D also support this observation [2,22,23].

It is conceivable that the WPM may have a positive biological effect on the function of beta-cells, potentially enabling them to better respond to elevated glucose levels, due to the longer exposure to WPM. Support from this observation stems from the acute study involving people with T2D [3], where the physiologic biphasic insulin pattern re-appeared with WPM, compared to the inert comparator, as well as its stimulating effect on both GLP-1 and glucagon response [3]. This however requires further confirmation.

The present study also provides insights into the effects of the relatively low dose of WPM on modulation of appetite and food consumption in both men and women. Previous studies have reported that the appetite-suppressing effects of WP pre-meals – and their influence on subsequent food intake – can vary depending on factors such as age, sex, and the amount of WP consumed [24,25], where typically higher doses have resulted in a more potent satiating effect [9]. One review even suggested that the satiety effects of WP could depend on body weight in a way that 50 g WP may suppress hunger in lean individuals but not people with overweight or obesity [26].

Possible mechanisms implicated in the reduction of appetite and food consumption seen may involve effects on GLP-1 and GIP, hormones that are secreted from gastrointestinal tract and affect appetite by regulating digestive processes and neural signaling in the central nervous system (CNS). WP also contain a high amount of amino acids, in particular branched chain amino acids, which for the WPM are made rapidly bioavailable [3], that can directly reduce the orexigenic neuropeptide, neuropeptide Y and increase anorexigenic neuropeptide, proopiomelanocortin in the hypothalamus [27,28]. Studies have suggested that key amino acids involved in this are isoleucine, leucine, lysine, methionine, phenylalanine, proline, tyrosine, and valine [11].

The effect on gastric emptying is also worth considering in this context, since a slower gastric emptying rate is associated with both a lower PPG and appetite suppression [10]. A previous study with WPM, as well with other WP formulation, have reported a modest reduction in gastric emptying rate [3,29]. The role of WPM in weight management for people with overweight or obesity should therefore be further studied, given the long history of safe use of WP, when these ingredients are taken alone or in conjunction with medications in humans, unless individuals have specific allergies to milk protein(s) or lactose. In this study we did not observe any serious adverse events related to WPM use.

## 5 Limitation

Although the cross-over design of the study minimizes inter-subject variability, and increases the internal validity of outcomes, limitations include the single-center and open-label design, a relatively small and homogeneous study population (all Caucasians), and a relatively short-term exposure. Also, glycemic assessments were performed with CGM, and not frequent blood sampling, although CGM is now accepted as a proxy for blood glucose levels. For the device used in the study, the mean absolute relative difference (MARD) between the CGM trace and the precise blood glucose concentration values, has been reported to be low (i.e., < 10 %), as also supported by a study comparing both arterialised and CGM values [4], providing reassurance. Furthermore, we did not assess effects on incretin hormones, insulin, or gastric emptying, and also the study was powered for glycaemic effects and not for appetite scoring. The latter may explain the less robust results on appetite due to more heterogenic effects on appetite scores. Finally, we did several analyses, where multiple testing was not corrected for, which may increase the chance of type II error, and hence further confirmatory studies are required to elucidate the satiety effects.

## 6 Conclusion

A 125 mL pre-meal beverage of a low dose WPM consumed twice daily 15 min ahead of breakfast and lunch for 4 days in people with overweight or obesity without diabetes, significantly reduced PPG during breakfast and had a moderate appetite suppressing effect, which led to a significantly lower amount of food consumed during subsequent ad-libitum lunch. This would suggest the potential of using a low dose premeal WPM in obesity management, although longer term, blinded, and larger studies with multicenter and multi-ethnic cohort-involvement of this novel nutraceutical are warranted.

Clinical take away messages:

• Existing evidence suggests that consuming pre-meal whey protein (WP) may have a moderate benefit on postprandial glucose and appetite in people with overweight or obesity, but few studies have assessed effects of lower doses of WP, consumed multiple times per day for several days when compared with a metabolically inert comparator.
• This study of a novel concentrated (125 mL), and low dose (10 g), non-gelling WP formulation (WP microgel [WPM]), consumed twice daily over 4 days, 15 min ahead of breakfast and lunch, found a significant benefit on reducing both postprandial glucose during a standardized breakfast meal, and mean 24 h glucose levels.
• Consumption of WPM also led to an appetite suppressing effect, which was associated with a significant but modest reduction in food consumption during an ad-libitum lunch meal.

## CRediT author statement

Conceptualization, O.E.J.; Methodology, I.J.N., K.T., B.A., R.J.C., A.M., E.M., and O.E.J.; Project administration, A.G., R.A., and O.E.J.; Funding acquisition, O.A., and O.E.J.; Formal analysis, E.M., and O.E.J., Investigation, I.J.N., B.A., A.M., and R.A.; Resources, O.A., and O.E.J.; Supervision, I.J.N., K.T., B.A., R.J.C., and O.E.J.; Writing—original draft preparation, O.E.J.; Visualization, O.E.J.; Writing—;review and editing, I.J.N., K.T., B.A., R.J.C., A.G., A.M., R.A., E.M., D.P., O.A., and O.E.J. All authors have read and agreed to the published version of the manuscript.

## Ethics review

This study was conducted in accordance with the Declaration of Helsinki and was approved on Aug 19, 2024 by the «Commission cantonale d’éthique de la recherche sur l’être humain du Canton de Vaud», with reference number 2024-00731. It was registered at clinicaltrials.gov with identifier: NCT06593769. All participants provided written informed consent.

## Declaration of Artificial Intelligence (AI) and AI-assisted technologies in the writing process

During the preparation of this work the authors did not use AI-assisted technologies.

## Source of funding

This study was funded by Nestlé Health Science, Vevey, Switzerland.

## Declaration of competing interest

Ian J Neeland has received speaker/consulting fees from Boehringer Ingelheim, Eli Lilly, Novo Nordisk, and Bayer Pharmaceuticals. Kostas Tsintzas has received study grants from Nestlé Health Science. Bo Ahrén has received lecture fees from Nestlé Health Science, USA, Mannkind Pharma, India, and Cipla Ltd, India., and owes stocks in Astra Zeneca, Eli Lilly and Novo Nordisk Pharmaceuticals. Robert J Chilton has received consulting fees/speaking honoraria from Medtronics, Boehringer Ingelheim, Lilly, and MSD. Ambra Giorgetti, Alric Mondragon, Rachel Ambiaux, and Eugenia Migliavacca are employees of Nestlé Research, Switzerland. David Phillipe, and Odd Erik Johansen are employees of Nestlé Health Science, Switzerland; Olivier Aprikian is an employee of Nestlé Health Science, US. The paper reflects the view of the scientists, and not the companies.
