# Supplementary material for: Short-term pre-meal whey protein microgel supplementation reduces postprandial glycemia and appetite in adults with overweight: An open-label randomised controlled trial
Source: Obes Pillars. 2025 May 26;15:100183. doi: 10.1016/j.obpill.2025.100183 (PMC12166780; doi:10.1016/j.obpill.2025.100183)
Supplement: Multimedia component 1 [file mmc1.docx]

**Supplementary material**

Glycemia and Appetite Effects of Short-term Twice Daily Pre-meal Very Low Dose Whey-protein Microgel in People with Overweight or Obesity without Diabetes – an Open-label Randomised Controlled Trial Ian J Neeland, Kostas Tsintzas, Bo Ahrén, Robert J Chilton, Ambra Giorgetti, Alric Mondragon, Rachel Ambiaux, Eugenia Migliavacca, David Phillipe, Olivier Aprikian, Odd Erik Johansen.

| **Section - page** | **Description** |
| --- | --- |
| Figure S1 - 2 | Study design and scheme |
| Table S1 - 3 | Study inclusion and exclusion criteria |
| Figure S2 - 4 | Illustration of timing of consumption of ready-to-drink pre-meal beverage (WPM or Control), standard breakfast or ad-libitum lunch, and VAS collection frequency. |
| Table S2 - 5 | Pre- and post-hoc defined analysis |
| Figure S3 - 6 | Consort Diagram |
| Figure S4 – 7 | VAS score: How satisfied do you feel? |
| Figure S5 – 8 | VAS score: How full do you feel? |
| Figure S6 – 9 | VAS score: How hungry do you feel? |
| Figure S7 – 10 | VAS score: How much do you think you could eat? |
| Table S3 – 11 | Adverse events overview |

**Figure S1. Study design and scheme**

**
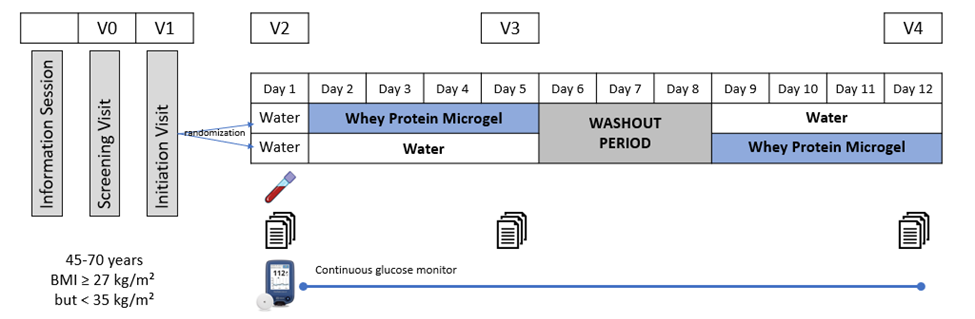
**

**Table S1. Inclusion and exclusion criteria for the study**

| Inclusion criteria |
| --- |
| 1. Males and females aged 45 – 70 years, at enrolment.  2. Body mass index (BMI) ≥ 27 kg/m² but < 35 kg/m².  3. Healthy participants based on medical history.  4. Able to understand and to sign a written informed consent prior to study enrolment.  5. Willing and able to comply with the requirements for participation in this study. |
| Exclusion criteria |
| 1. Any past or on-going diagnosed medical/surgical condition (i.e., diabetes, malignancy, gastrointestinal disease, chronic inflammatory condition, chronic kidney disease, cardiovascular disease, bleeding disorder) and/ or psychiatric condition (e.g. depression, psychotic disorders, chronic insomnia, eating disorder), which in the opinion of the site physician/investigator may risk participant’s well-being/safety, impede participant compliance with study procedures or ability to complete the study and/or could confound the primary objectives of the study.  2. Random plasma glucose ≥ 11.1 mmol/L or fasting plasma glucose ≥ 7.0 mmol/L (finger-prick point-of-care testing).  3. HbA1c ≥ 6.5% (finger-prick point-of-care testing).  4. Known inborn errors of amino acid and protein metabolism.  5. With planned/scheduled medical imaging procedure/s (magnetic resonance imaging [MRI], computed tomography [CT] scan, X-ray), or electrical heat (diathermy) treatment during the study period.  6. Known or suspected allergies or intolerances to any of the ingredients of the nutritional formulation (i.e., milk, lactose) and the study meals.  7. Known or suspected cutaneous hypersensitivity to adhesives, silicon, or plaster.  8. Substantial changes in eating habits (i.e., switching from one dietary regimen to another) up to 30 days before enrolment.  9. Anticipated change in usual physical activity levels during the study period (e.g., plans for undertaking new vigorous-intensity physical activity such as: jogging, running, carrying heavy objects or other loads upstairs, shoveling snow, participating in a fitness class, and fast swimming, that will lead to hard and fast breathing).  10. Any chronic alcohol or drug abuse within the past year; specifically, alcohol intake > 2 servings per day for males and >1 serving per day for females. A serving corresponds to 0.35dl of liquor/strong alcohol, 1 dl of wine, or 3 dl of beer.  11. Female participants who are pregnant, lactating and/or breastfeeding.  12. Participants who refuse to be informed of incidental findings relevant to their health (e.g. abnormal laboratory results).  13. Currently participating in another interventional study.  14. Family or hierarchical relationships with the research team members. |

**Figure S2** lllustration of timing of consumption of ready-to-drink pre-meal beverage (WPM or Control), standard breakfast or ad-libitum lunch, and VAS collection frequency.


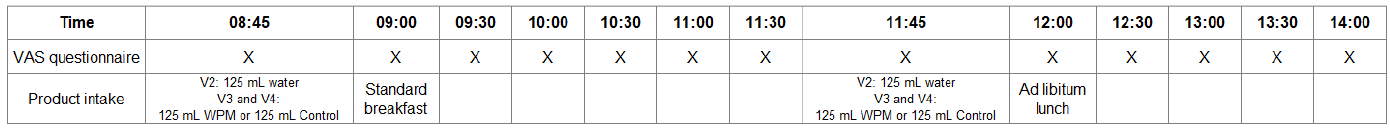


Abbreviations: WPM – whey protein microgel; VAS – visual analogue scale

**Table S2.** Pre- and post hoc defined analysis

|  | **Predefined** | **Post-hoc defined** |
| --- | --- | --- |
| Glucose | Clinic PPG iAUC -15-120 min during standardized breakfast meal  Clinic PPG iAUC -15-120 min during ad-libitum lunch meal  3-days of free-living mean glucose 24h CGM |  |
| Appetite | VAS scores iAUC -15 min – 165 min (breakfast)  VAS scores iAUC 165 min – 300 min (lunch) | Average VAS scores breakfast meal occasion (0-165 min)  Average VAS score lunch meal occasion (180-300 min)  VAS scores at induvial time-points  VAS scores tAUC -15 min – 165 min (breakfast)  VAS scores tAUC 165 min – 300 min |
| Food consumption | Amount of food consumed during ad-libitum pizza lunch |  |

Abbreviations: iAUC – incremental area under the curve; tAUC – total area under the curve; PPG – postprandial glucose; VAS – visual analogue scale

**Figure S3. Consort diagram** Abbreviations: BID – twice daily; CTR – control; WPM – whey protein microgel


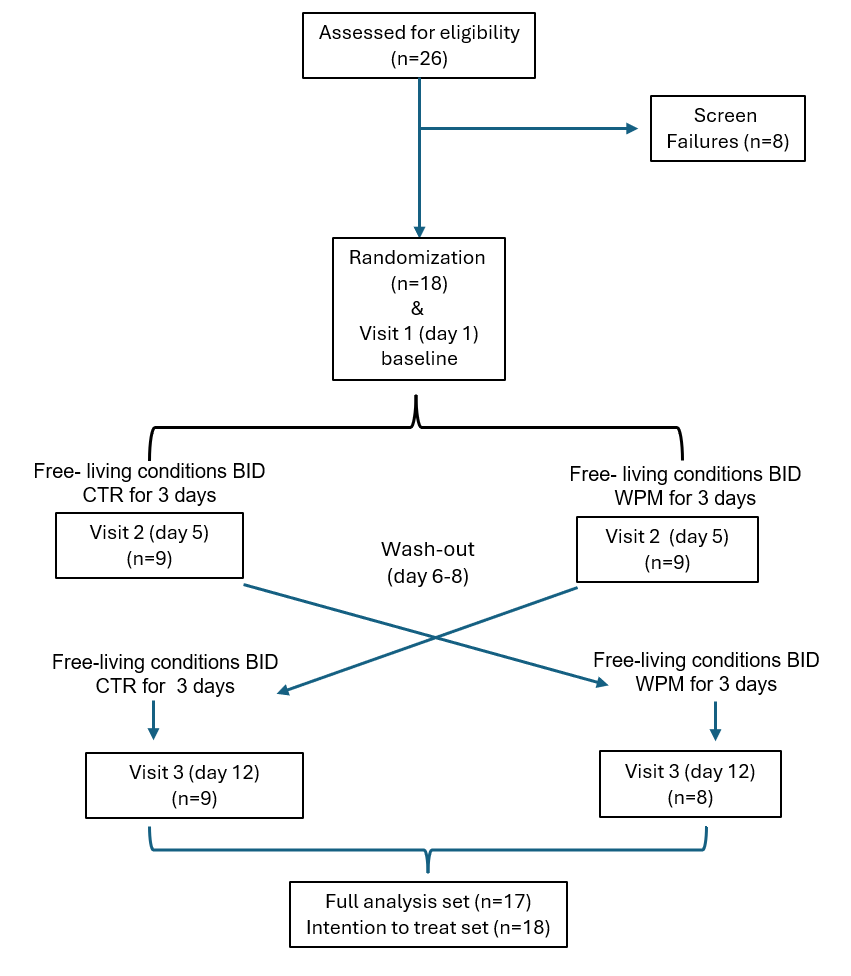


**Figure S4** VAS responses to dimension “How satisfied do you feel?” over standardized breakfast and ad-libitum lunch preceded by premeal (-15 min) WPM or control.

Abbreviations: iAUC – incremental area under the curve, PBO – placebo, WPM, whey protein microgel, SE – standard error, h - hours.

**Figure S5** VAS responses to dimension “How full do you feel?” over standardized breakfast and ad-libitum lunch preceded by premeal (-15 min) WPM or control.

Abbreviations: iAUC – incremental area under the curve, PBO – placebo, WPM, whey protein microgel, SE – standard error, h - hours.

*: p<0.05.

**Figure S6** VAS responses to dimension “How hungry do you feel?” over standardized breakfast and ad-libitum lunch preceded by premeal (-15 min) WPM or control.

**Figure S7** VAS responses to dimension “How much do you think you could eat?” over standardized breakfast and ad-libitum lunch preceded by premeal (-15 min) WPM or control.

**Table S3.** Adverse events overview

| SOC term | PT term | Total cases |
| --- | --- | --- |
| Ear and labyrinth disorders | Motion sickness | 1 |
| Gastrointestinal disorders | Abdominal pain  Nausea | 1  1 |
| Infections and infestations | Influenza  Nasopharyngitis  Pneumonia | 2  1  1 |
| Nervous system disorders | Dizzines  Headache | 1  1 |
| Respiratory, thoracic and mediastinal disorders | Oropharyngeal pain | 1 |
| Vascular disorders | Varicose vein | 1 |

| AE by consumption period | Events | Subjects |
| --- | --- | --- |
| Pre-consumption period (after V0, but before V1) | 4 | 4 |
| WPM consumption period | 7 | 3 |
| CTR consumption period | 0 | 0 |
